# Antimicrobial Archetypes: Assessing the Knowledge, Attitude, and Practice (KAP) Trends in Antimicrobial Resistance (AMR) and Antimicrobial Stewardship Program (AMSP) Among Faculties, Residents, and Interns in a Tertiary Care Hospital

**DOI:** 10.7759/cureus.64722

**Published:** 2024-07-17

**Authors:** Lavanya Balaji, Abiramasundari V K, Manivannan Nandhagopal, Jayakumar Subramaniam

**Affiliations:** 1 Department of Microbiology, Saveetha Medical College and Hospitals, Saveetha Institute of Medical and Technical Sciences, Saveetha University, Chennai, IND

**Keywords:** antimicrobial stewardship practices (amsp), amr awareness, kap questions, antimicrobial stewardship program, antimicrobial resistance

## Abstract

Background

Antimicrobial resistance (AMR) is caused by inappropriate use of antimicrobials. India's high antibiotic use contributes significantly to AMR. Antimicrobial Stewardship Programs (AMSPs) are crucial for optimizing antimicrobial use. Knowledge, Attitude, and Practice (KAP) studies are essential for evaluating healthcare professionals' beliefs and conduct regarding AMR and AMSPs.

Materials and methods

A cross-sectional study at Saveetha Medical College and Hospital evaluated doctors' knowledge, attitudes, and practices regarding AMR and stewardship programs. The study involved 202 participants, including faculty members, postgraduates, and interns.

Results

The study involved 202 participants, with residents being the majority at 51.4%, followed by faculty at 26.7% and interns at 21.7%. Faculty members showed the highest overall knowledge, followed by residents and interns. Despite possessing knowledge, practitioners did not consistently implement their knowledge in their daily practices, with a statistically significant difference of p < 0.01. There was a substantial disparity in attitude between the departments, as evidenced by a statistically significant p-value of less than 0.01.

Conclusion

Positive trends in knowledge and attitudes exist, but there are areas for improvement in translating attitudes into clinical practices. There is a significant disparity among faculty members, residents, and interns, highlighting the urgent need for interventions to bridge the gap. Implementing antibiotic prescribing guidelines at the institutional level and enhancing knowledge, attitudes, and practices among healthcare professionals are crucial to addressing AMR.

## Introduction

Antimicrobial resistance (AMR) is a widespread phenomenon where microorganisms develop resistance to antimicrobial agents, making them ineffective in treating infections [1]. It is caused by genetic mutations or acquiring resistance genes through horizontal gene transfer, enabling microorganisms to neutralize the antimicrobial activity of drugs designed to curb them [2]. This leads to increased use of broad-spectrum empiric antibiotic therapy, narrowing treatment options, and worsening patient outcomes. India leads the world in human antibiotic use, with 10.7 units per person, significantly driving AMR [2,3].

AMR is primarily driven by the overuse and inappropriate use of antimicrobials, insufficient infection prevention and control practices, and extensive use of antimicrobials in agriculture and food production. Over-the-counter use of antibiotics, overpopulation, lack of awareness, inadequate use of diagnostics, cross-infections, and poor health infrastructure amplify India's AMR problem [4,5]. The emergence of resistance-related infections leads to higher levels of morbidity, mortality, and healthcare expenses, as patients may need prolonged hospital stays, more intensive medical interventions, and alternative, often more expensive, antimicrobial therapies.

According to estimates from the World Bank, AMR could result in an additional US$1 trillion in healthcare costs by 2050 and between US$1 trillion and US$3.4 trillion in gross domestic product (GDP) losses per year by 2030 [1,6]. Furthermore, AMR hinders the effectiveness of standard medical procedures such as surgery, chemotherapy, and organ transplantation, as the presence of resistant pathogens raises the risk of postoperative infections and treatment setbacks [7]. Bacterial AMR is estimated to have directly caused 1.27 million deaths worldwide in 2019 and contributed to 4.95 million deaths. Addressing AMR is crucial to maintaining the safety and efficacy of medical interventions and ensuring positive patient outcomes [1].

Antimicrobial stewardship programs (AMSPs) have become an essential tool in combating the growing problem of AMR. These programs are designed to optimize the use of antimicrobial agents, mitigate resistance, and preserve the effectiveness of available antimicrobial agents [8,9]. To achieve this, AMSPs use a multifaceted approach that includes surveillance, education, guideline development, and intervention strategies. By promoting judicious antimicrobial prescribing practices, AMSPs aim to minimize the emergence and spread of resistant pathogens, reduce antibiotic expenditures, shorten hospital stays, lower the incidence of adverse events associated with antimicrobial use, and improve patient outcomes [10]. Therefore, AMSPs are an indispensable tool in the battle against AMR, essential to any comprehensive public health strategy.

Healthcare professionals must implement effective AMSPs in their hospitals to ensure optimal patient outcomes and promote the judicious use of antibiotics [11]. Knowledge, Attitude, and Practice (KAP) studies play a crucial role in evaluating healthcare professionals' beliefs, understanding, and conduct concerning AMR and AMSPs. These studies help identify gaps in knowledge, misconceptions, and obstacles to practical implementation. By recognizing these factors, customized interventions can be developed to increase awareness of AMR, promote the proper use of antibiotics, and improve adherence to AMSP guidelines. This study was conducted among Saveetha Medical College faculty, residents, and interns to assess their knowledge of AMR and their practices regarding AMSPs.

## Materials and methods

Study setting and design

A cross-sectional analytical study was conducted among faculty members, residents, and interns of Saveetha Medical College and Hospital in Thandalam, Tamil Nadu, India, to assess doctors' knowledge, attitudes, and practices regarding AMR and AMSPs. The study included 202 respondents and was conducted from July 2023 to August 2023. Analysis was performed between September 2023 and October 2023.

The study was conducted after obtaining ethical clearance from our hospital's institutional review board. The participants in the questionnaire referenced in this article provided consent in accordance with ethical standards and procedures. They were informed about the study's purpose, role, and rights as research subjects. It is important to note that the requirement for written consent was obtained and waived for this study.

Inclusion criteria

Faculty members, postgraduates, and interns from Saveetha Medical College and Hospital were included in the study.

Exclusion criteria

First-year, second-year, third-year, and final-year MBBS students were excluded from the study.

Data collection and questionnaire development

After obtaining informed consent, the study participants were asked to complete a pre-validated structured questionnaire consisting of 12 questions that focused on AMR and stewardship programs. This questionnaire was meticulously created, drawing upon extensive literature reviews, and distributed in four sections (Demographics, Knowledge, Attitude, and Practices) via Google Forms to ensure an efficient and streamlined process. The responses to the questions were recorded on a three-point Likert Scale with options as Agree, Disagree, and No Comment [12]. The questionnaire was distributed to interns, residents, and faculty to gather information about their knowledge of AMR, attitude toward prescription habits, agreement or disagreement with certain perceptions regarding antibiotics, preferred selection of antibiotics in specific settings, and suggestions for rationalizing the use of antimicrobial medication in their practice. Only the participants who completed the survey in total were included in the analysis.

Data management

The data were collected through Google Forms and analyzed using statistical methods. For quantitative data, the mean and standard deviation were calculated. The categorical data were computed as percentages, and the chi-square test (χ² test) was performed to look for statistical significance. A significance level of p < 0.05 was considered statistically significant. The correlation between various sociodemographic factors and respondents' knowledge, attitudes, and practices regarding the usage and resistance of antibiotics was also analyzed. The results are presented in tables and graphs that protect the anonymity of the participants.

Assessment index

The questionnaire employs a three-point Likert scale to record responses as Agree, No Comment, or Disagree, with each response assigned a score (Agree, 2 points; No Comment, 1 point; Disagree, 0 points). The scores are then tallied for each section, ranging from 0 to 8. Respondents who score six or higher in each section are considered to have good knowledge, a favorable attitude, and good practice, respectively. A cutoff score above 60% in the Knowledge section represents a comprehensive understanding of AMR and AMSPs, while scores below 40% indicate a poor grasp of these concepts. In the Attitude section, scores above 60% demonstrate a positive outlook, while scores below 40% reflect an unfavorable attitude. Similarly, scores above 60% in the Practice section indicate better adherence to recommended practices, while scores below 40% suggest poor compliance [13]. Integrated scores from all three sections provide a comprehensive evaluation of knowledge, attitudes, and practices regarding AMR and AMSPs. 

The scores of the faculty, residents, and interns' knowledge, attitude, and practices have been compiled and classified as illustrated in Appendix 1. The scores on the knowledge, attitude, and practices of critical care medicine, medical and medical subspecialties, and surgical and surgical subspecialties have been compiled and classified as illustrated in Appendix 2. The questionnaire is included in Appendix 3.

## Results

Characteristics of the respondents

Table 1 provides an overview of the demographics of the participants who participated in the study. Two hundred two participants completed the survey, resulting in an impressive response rate of 82.4% (n=202). The respondents were categorized into faculties, residents, and interns as per their designation, with medical sub-specialties, surgical and surgical sub-specialties, and critical medicine departments as their primary specialties.

**Table 1:** Demographics of the respondents.

Characteristics	Number (n= 202)	Percentage (n %)
Primary Specialty		
Medical and medical sub-specialties	90	44.5
Surgical and surgical sub-specialties	79	39.1
Critical care medicine	33	16.3
Designation		
Faculty	54	26.7
Residents	104	51.4
Interns	44	21.7

**Figure 1:**
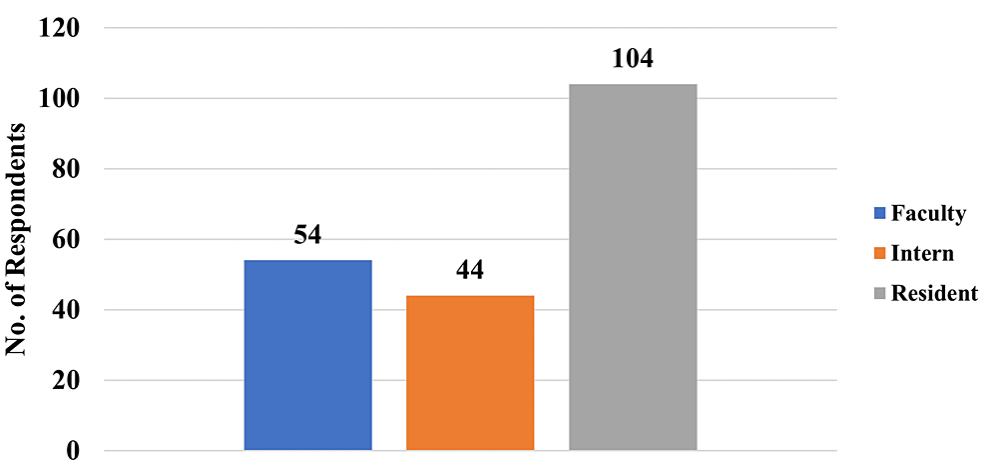
Distribution of respondents as per their designation.

The respondents were primarily residents, accounting for 51.4% (n=104) of the total, followed by faculty members at 26.7% (n=54), and interns at 21.7% (n=44), as shown in Figure 1. 

**Figure 2:**
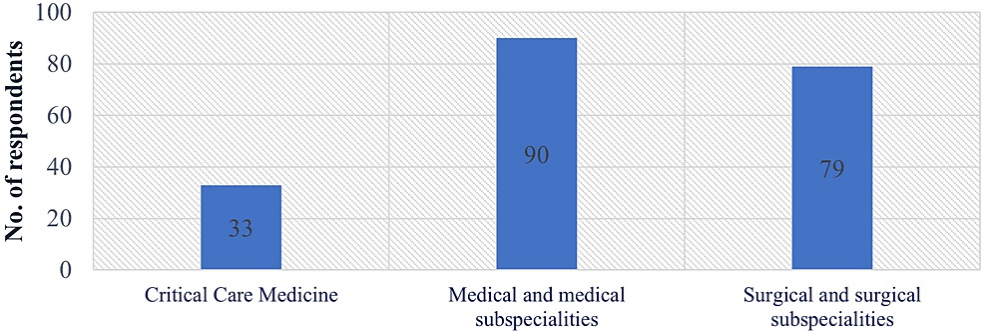
Distribution of respondents as per their primary specialties.

The Medical and Medical sub-specialties recorded the highest response rate of 44.5% (n=90), followed by Surgical and Surgical sub-specialties at 39.1% (n=79) and Critical Care Medicine at 16.3% (n=33), as shown in Figure 2. 

**Figure 3:**
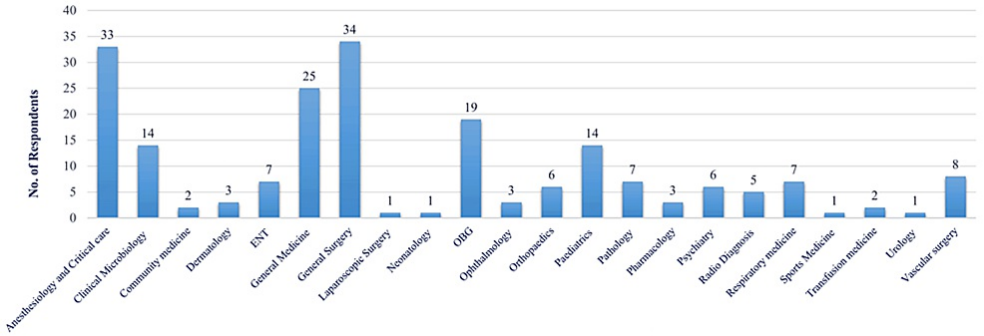
Distribution of respondents as per their departments.

The distribution of respondents as per their departments has been computed into a graph, as shown in Figure 3.

In the Knowledge section, there were five questions for assessing the participants' understanding of the prevalence of resistance, the duration of antimicrobial treatment, and the factors contributing to drug resistance. The results showed that 90% (n=182) of the participants agreed that changes in antimicrobial use are correlated with the prevalence of resistance, while 1.98% (n=4) disagreed. Additionally, 85.6% (n=173) of the participants acknowledged that AMR is more common in hospitals than in the community, especially in patients who have previously received antimicrobials, whereas 5.4% (n=11) did not. A significant majority, around 92.5% (n=187), believed that patients exposed to antimicrobials for longer or shorter durations than necessary are at increased risk of developing AMR. However, only 40.5% (n=82) of the participants disagreed that antibiotic restrictions in hospitals would negatively impact patient care because doctors would not be able to prescribe antibiotics as they see fit.

In the Attitude section, there were five questions that evaluated the participants' attitudes toward optimizing drug selection, dosage, duration, and therapy route. When suspecting an infection, 58.4% (n=118) of the participants disagreed with immediately starting antibiotic therapy before sending the appropriate culture, while 37.6% (n=76) agreed. Additionally, 95.5% (n=193) of the respondents expressed that they would switch to a suitable antibiotic after receiving the culture and sensitivity report, whereas 2.4% (n=5) disagreed with this view. Furthermore, 86.3% (n=173) of the respondents stated that they would review their patients after 48-72 hours of initiating therapy, while 9.4% (n=19) did not. Finally, 86.3% (n=175) of the participants indicated confidence in their ability to initiate the right antimicrobial choice, dose, and route of administration, and to adjust the therapy based on clinical and diagnostic evaluation, whereas 5.9% (n=12) did not.

In the Practice section, a different set of five questions was used to assess the respondents' day-to-day practices regarding antibiotic selection, adherence to prescription habits, and stewardship policies. It was found that 86.3% (n=175) of the respondents reported practicing de-escalation of antimicrobials from parenteral therapy to oral therapy when it is deemed necessary to reduce AMR, while 7.9% (n=16) disagreed with this practice. Only 36.6% (n=74) of the respondents agreed with discontinuing antibiotics after a no-growth report, with a notable 53.4% (n=108) stating that they do not stop the antibiotic after a no-growth report. Furthermore, 85.6% (n=173) of the participants indicated adherence to stewardship policies in their hospital, while 9.4% (n=19) did not. Additionally, 66.8% (n=138) of the respondents reported that their choice of antimicrobials is based on the AWaRe (ACCESS, WATCH, AND RESERVE) classification, while 15.3% (n=31) disagreed.

Based on the overall findings from Table 2, most participants, accounting for 79% (n=160), displayed a good understanding of the factors contributing to AMR. On the other hand, 15.5% (n=42) of the respondents disagreed with this notion. Moreover, 81.6% (n=164) of all participants expressed a positive attitude toward optimizing drug selection, dosage, duration, and therapy route, while 14.2% (n=38) did not. Additionally, a significant number of the participants, 68.8% (n=139), demonstrated good practices in selecting antibiotics and adhering to prescription habits and stewardship policies, while 20.8% (n=63) did not express such practices.

**Table 2:** Respondents' responses to the Knowledge, Attitude, and Practice questionnaire on antimicrobial resistance and antimicrobial stewardship programs. AMR: antimicrobial resistance; AMSP: antimicrobial stewardship program.

Questions/statements	Responses (n %)		
	Agree	Disagree	No comment
Knowledge of AMR and AMSP			
Changes in antimicrobial use are paralleled by the prevalence of resistance	182 (90)	4 (1.98)	16 (7.9)
Antimicrobial resistance is more prevalent in a hospital than in a community and in patients who have received prior antimicrobials	173 (85.6)	18 (8.9)	11 (5.4)
Patients exposed to longer/shorter duration of antimicrobials than necessary have an increased risk of developing antimicrobial resistance	187 (92.5)	11 (5.4)	4 (1.98)
Antibiotic restrictions in a hospital will negatively impact patient care because doctors cannot prescribe antibiotics, they want	92 (45.5)	82 (40.5)	28 (13.8)
Attitude on AMR and AMSP			
In case of suspecting an infection, I will immediately start antibiotic therapy before sending the appropriate culture	76 (37.6)	118 (58.4)	8 (3.9)
I’ll change to a suitable and appropriate antibiotic of choice after receiving the culture and sensitivity report	193 (95.5)	5 (2.4)	4 (1.98)
In an inpatient setting, I’ll review my patient after 48-72 hours of starting therapy	175 (86.3)	19 (9.4)	8 (3.9)
In an inpatient setting, I’m confident in my ability to put the patient on the right antimicrobial of choice, dose, and route of administration and change the therapy to targeted antibiotic choice (deescalate) according to clinical and diagnostic evaluation	175 (86.3)	15 (7.4)	12 (5.9)
Practices on AMR and AMSP			
In an inpatient setting, I adhere to stepping down the antimicrobials from para-enteral therapy to oral therapy if it is necessary to reduce antimicrobial resistance	175 (86.3)	11 (5.4)	16 (7.9)
In case of a “No Growth” in the culture report, I’ll immediately stop the antibiotic	74 (36.6)	108 (53.4)	20 (9.9)
I prescribe antimicrobials adhering to the AMSP followed in our hospital	173 (85.6)	19 (9.4)	10 (4.9)
My selection of antimicrobials is based on AWaRe classification of antibiotics (ACCESS, WATCH, AND RESERVE group)	135 (66.8)	31 (15.3)	36 (17.8)

The participants' performances were evaluated according to the assessment index outlined in the materials and methods section, with scores ranging from 0 to 8 for each section. Those who achieved a score of six or higher in each section were deemed to possess good knowledge, a positive attitude, and good practice, respectively. The average scores for knowledge and attitude were approximately 6.52 and 6.73, indicating a favorable level, while the mean practice score was only 5.84, reflecting a lower standard of practice. The mean, median scores, and standard deviation of knowledge, attitude, and practices among the respondents have been computed in Table 3.

**Table 3:** Mean, median, and standard deviation of the respondents' score to the questionnaire.

	Knowledge	Attitude	Practice
Mean	6.52	6.73	5.84
Median	6.00	6.00	6.00
Standard deviation	±1.246	±1.340	±1.770

KAP trends regarding AMR and AMSP among faculties, residents, and interns

The present study assessed the knowledge, attitudes, and practices of faculty members, residents, and interns regarding AMR and optimization of drug dose and duration. Findings indicated that faculty members exhibited the highest level of overall knowledge at 90.7% (n=49), followed by residents at 84% (n=88) and interns at 72.4% (n=32). Notably, 90.7% (n=49) of faculty members, 86.7% (n=90) of residents, and 83% (n=36) of interns displayed a favorable attitude toward optimizing the selection of drugs, dosage, and duration and route of therapy. However, a discrepancy was observed between knowledge and practice across all three groups. Despite possessing good knowledge, the respondents' practices did not align with their understanding, with a statistically significant difference of p < 0.01. In this regard, faculty members demonstrated the highest reasonable practice rate at 70.3% (n=39), followed by residents at 63.3% (n=65). However, interns were found to have the least compliance with prescription habits at 61.1% (n=27).

KAP trends regarding AMR and AMSP among primary specialties

The survey results indicate that respondents from Medical and Medical Subspecialties had an impressive overall knowledge of AMR, with a rate of 92.2% (n=83). Surgical and surgical subspecialties followed this at 81% (n=64) and critical care medicine at 75.8% (n=25). Regarding attitudes toward optimizing drug selection, stepping down from parenteral to oral route, and switching from broad to narrow spectrum, the highest levels were observed in Medical and Medical Subspecialties, at 90% (n=81). This was followed by critical care medicine at 87.9% (n=29) and surgical specialties at 86.1% (n=68). Adherence to prescription practices and stewardship policies was high in surgical and surgical subspecialties, at 70% (n=55). Medical and Medical Subspecialties followed at 62.2% (n=56), while the least compliance was seen in critical care medicine, at 57.6% (n=19). 

Despite possessing knowledge in all primary specialties, the practitioners did not consistently implement this knowledge in their daily practices. Additionally, there was a significant disparity in attitude between the three departments, as evidenced by a statistically significant p-value of less than 0.01.

## Discussion

Our study presented a comprehensive evaluation of KAP regarding AMR and AMSP among healthcare professionals, including faculty members, residents, and interns, across different primary specialties. The findings shed light on both commendable aspects and areas needing improvement within healthcare settings. Understanding these aspects is crucial for effectively combating the global threat AMR poses.

Our study showed that 79% (n=160) of the respondents demonstrated commendable levels of knowledge, showing a good understanding of the factors contributing to AMR. This indicates a positive trend in awareness, which is crucial for effective interventions. However, it is concerning that 15.5% (n=42) of our respondents disagreed with certain statements related to AMR, indicating gaps and challenges in the implementation of such policies. Similar findings have been reported in studies conducted by Wester et al. and Al-Harthi et al. among clinicians, where the clinicians showed overall good knowledge regarding antibiotic resistance, which is encouraging as better knowledge is often linked to better health practices [14,15]. However, efforts should be made to address these misconceptions through targeted educational initiatives and continuous professional development programs.

Our study found that over 81.6% (n=164) of the participants showed a positive attitude toward optimizing drug selection, dosage, duration, and therapy route. This suggests that healthcare professionals are willing to adopt practices that combat AMR and improve patient care outcomes. Interestingly, interns and residents in training demonstrated less confidence in antimicrobial prescribing than faculty members. This aligns with a study by Garcia et al., where residents showed less confidence in prescribing antibiotics [16]. Another study by Srinivasan et al. also reported a similar trend, with senior residents being more optimistic about the appropriate use of antimicrobials than first-year residents [17]. However, it is essential to consider the perspectives of the minority at 14.2% (n=38) who did not express positive attitudes, as their viewpoints could impact clinical decision-making processes.

Our study uncovered a notable disparity between knowledge and practical application across all participant cohorts within their respective practices, demonstrating a statistically significant variance (p < 0.01). Despite possessing sound knowledge and favorable attitudes, participants did not consistently translate these principles into action, particularly evident among interns, with a practice rate of 68.8% (n=139). This discrepancy underscores the imperative for targeted interventions to rectify this issue. Our study's findings were similar to those conducted by Firouzabadi et al. and Babatola et al., where the knowledge of healthcare workers did not translate into their daily practices [18,19]. Furthermore, the disparities in adherence to prescription practices among faculty members, residents, and interns underline the necessity for tailored educational interventions to address each group's distinct needs and challenges.

The research also investigated the KAP trends across different primary specialties, uncovering disparities in understanding, attitudes, and behaviors among survey participants. Notably, respondents from medical and medical subspecialties demonstrated a commendable overall understanding of AMR. However, participants in critical care medicine exhibited poor compliance with prescription practices and stewardship policies (p < 0.01). Similar findings were observed in the study by Chatterjee et al. [20], where despite having satisfactory background knowledge about the rational use of antimicrobials and AMR patterns, discrepancies were noted in the physicians’ prescribing attitudes. Moreover, respondents in medical and medical subspecialties achieved higher overall KAP scores than their surgical counterparts, aligning with previous findings in an Indian survey [20,21]. These results underscore the necessity of tailoring educational interventions and stewardship programs to address each specialty's distinct challenges and priorities.

Limitations of the study

Our study primarily included healthcare professionals from a specific institution or region. This could limit how well the results apply to a larger population. The data were based on self-reported responses, which could be influenced by people wanting to give socially acceptable answers. This might have led to biased or exaggerated results. The study's accuracy could be affected by the reliability and validity of the questionnaire to assess knowledge, attitudes, and practices related to AMR. The findings might not apply directly to healthcare settings with different organizational structures, resources, and patient populations since the focus was on a single institution.

## Conclusions

In conclusion, this study's findings provide valuable insights into the knowledge, attitude, and practice regarding AMR and AMSP among medical professionals. While there are positive trends in knowledge and attitudes, there are also areas for improvement, particularly in translating attitudes into clinical practices irrespective of their department or designation. The disparity was statistically significant even among faculty members, residents, and interns. This highlights the urgent need for interventions to bridge the gap and help healthcare providers use their knowledge optimally while prescribing drugs. Establishing continuous medical education programs and developing and monitoring institutional antibiotic policies are essential to address this issue. To implement AMSPs effectively, it is crucial to involve infectious disease consultants, clinical microbiologists, hospital infection control nurses, and pharmacists. Implementing antibiotic prescribing guidelines at the institutional level and using additional measures such as formulary restriction is crucial to address the knowledge-attitude dissonance identified in the survey. By enhancing KAP among healthcare professionals, we can work towards mitigating the threat of AMR and ensuring the effective use of antimicrobial agents in clinical practice.

## Appendices

Appendix 1

Scores of Knowledge, Attitude, and Practice among faculty, interns, and residents are shown in Table 4.

**Table 4:** Scores of Knowledge, Attitude, and Practice among faculty, interns, and residents. The data are presented as N (%). The p-value is calculated using the chi-square test. p-value <0.05 is considered statistically significant.

Variable	Score	Faculty	Intern	Resident	p-value
Knowledge	2	1(50.0%)	1(50.0%)	0(0%)	0.323
	4	4(40.0%)	0(0%)	6(60.0%)	
	5	0(0%)	9(15.8%)	9(9.6%)	
	6	23(28.0%)	18(22.0%)	41(50.0%)	
	7	6(28.6%)	5(23.8%)	10(47.6%)	
	8	20(35.1%)	3(25.0%)	28(49.1%)	
Attitude	2	0(0%)	1(50%)	1(50%)	0.397
	3	1(50%)	1(50%)	0(0%)	
	4	2(20.0%)	2(20.0%)	6(66.7%)	
	5	2(33.3%)	2(33.3%)	6(60.0%)	
	6	16(21.6%)	14(18.9%)	44(59.5%)	
	7	3(33.3%)	0(0%)	2(33.4%)	
	8	30(37.0%)	16(19.8%)	35(43.2%)	
Practice	0	1(100%)	0(0%)	0(0%)	0.001
	2	1(12.5%)	0(0%)	7(7.4%)	
	3	6(100%)	0(0%)	0(0%)	
	4	4(28.6%)	6(18.8%)	20(21.3%)	
	5	4(18.2%)	8(15.4%)	7(50.0%)	
	6	17(31.5%)	2(9.1%)	27(51.9%)	
	7	6(18.8%)	3(21.4%)	16(72.7%)	
	8	15(30.6%)	17(34.7%)	17(34.7%)	

Appendix 2

Scores of KAP among critical care medicine, medical and medical subspecialties, surgical and surgical subspecialties, and critical medicine are shown in Table 5.

**Table 5:** Scores of Knowledge, Attitude, and Practice among critical care medicine, medical and medical subspecialties, surgical and surgical subspecialties, and critical medicine. The data are presented in N (%). The p-value is calculated using the chi-square test. p-value <0.05 is considered statistically significant.

Variable	Score	Critical care medicine	Medical and medical subspecialties	Surgical and surgical subspecialties	p-value
Knowledge	2	0(0%)	1(50%)	1(50%)	0.064
	4	0(0%)	3(30.0%)	7(70.0%)	
	5	6(50%)	1(8.3%)	3(25.0%)	
	6	11(13.4%)	42(51.2%)	23(38.3%)	
	7	3(14.3%)	9(42.9%)	7(33.3%)	
	8	8(14.0%)	26(45.6%)	19(33.3%)	
Attitude	2	0(0%)	1(50%)	0(0%)	0.001
	3	0(0%)	1(50%)	0(0%)	
	4	1(11.1%)	4(44.4%)	4(44.4%)	
	5	1(16.7%)	1(16.7%)	3(50%)	
	6	15(20.3%)	20(27.0%)	35(47.3%)	
	7	3(30.0%)	5(50%)	0(0%)	
	8	8(28.6%)	50(61.7%)	18(22.2%)	
Practice	0	0(0%)	1(100%)	0(0%)	0.759
	2	2(25.0%)	5(62.5%)	1(12.5%)	
	3	0(0%)	2(33.3%)	4(66.7%)	
	4	5(15.6%)	15(46.9%)	9(28.1%)	
	5	5(22.7%)	9(40.9%)	6(27.3%)	
	6	8(15.4%)	25(48.1%)	17(32.7%)	
	7	4(28.6%)	4(28.6%)	4(28.6%)	
	8	4(8.2%)	21(42.9%)	19(38.8%)	

Appendix 3

The Knowledge, Attitude, and Practice (KAP) questionnaire on antimicrobial resistance (AMR) and antimicrobial stewardship program (AMSP).

**Table 6:** The Knowledge, Attitude, and Practice (KAP) questionnaire. AMR: antimicrobial resistance, AMSP: antimicrobial stewardship program.

Questions/statements
Knowledge of AMR and AMSP
1. Changes in antimicrobial use are paralleled by the prevalence of resistance
a) Agree
b) Disagree
c) No comment
2. Antimicrobial resistance is more prevalent in a hospital than in a community and in patients who have received prior antimicrobials
a) Agree
b) Disagree
c) No comment
3. Patients exposed to longer / shorter durations of Antimicrobials than necessary have an increased risk of developing Antimicrobial resistance
a) Agree
b) Disagree
c) No comment
4. Antibiotic restrictions in a hospital will negatively impact patient care because doctors cannot prescribe antibiotics; they want
a) Agree
b) Disagree
c) No comment
Attitude on AMR and AMSP
1. In case of suspecting an infection, I will immediately start antibiotic therapy before sending the appropriate culture:
a) Agree
b) Disagree
c) No comment
2. I’ll change to a suitable and appropriate antibiotic of choice after receiving the culture and sensitivity report
a) Agree
b) Disagree
c) No comment
3. In an inpatient setting, I’ll review my patient after 48-72 hours of starting therapy
a) Agree
b) Disagree
c) No comment
4. In an inpatient setting, I’m confident in my ability to put the patient on the right Antimicrobial of choice, dose, and route of administration and change the therapy to targeted antibiotic choice (deescalate) according to clinical and diagnostic evaluation.
a) Agree
b) Disagree
c) No comment
Practices on AMR and AMSP
1. In an inpatient setting, I adhere to stepping down the Antimicrobials from para-enteral therapy to oral therapy if it is necessary to reduce antimicrobial resistance
a) Agree
b) Disagree
c) No comment
2. In case of a “No Growth” in the culture report, I’ll immediately stop the antibiotic
a) Agree
b) Disagree
c) No comment
3. I prescribe antimicrobials adhering to the AMSP followed in our hospital
a) Agree
b) Disagree
c) No comment
4. My selection of antimicrobials is based on AWaRe classification of antibiotics (ACCESS, WATCH, AND RESERVE group)
a) Agree
b) Disagree
c) No comment
